# Cytotoxic Proteins and Therapeutic Targets in Severe Cutaneous Adverse Reactions

**DOI:** 10.3390/toxins6010194

**Published:** 2014-01-03

**Authors:** Shih-Chi Su, Wen-Hung Chung

**Affiliations:** 1 Department of Dermatology, Drug Hypersensitivity Clinical and Research Center, Chang Gung Memorial Hospitals, Taipei, Linkou, and Keelung, 33305, Taiwan; E-Mail: zenith5862@hotmail.com; 2 College of Medicine, Chang Gung University, Taoyuan 33302, Taiwan

**Keywords:** Stevens-Johnson syndrome, toxic epidermal necrosis, granulysin, perforin, granzyme B, Fas/Fas ligand

## Abstract

Severe cutaneous adverse reactions (SCARs), such as Stevens-Johnson syndrome (SJS) and toxic epidermal necrosis (TEN), are rare but life-threatening conditions induced mainly by a variety of drugs. Until now, an effective treatment for SJS/TEN still remains unavailable. Current studies have suggested that the pathobiology of drug-mediated SJS and TEN involves major histocompatibility class (MHC) I-restricted activation of cytotoxic T lymphocytes (CTLs) response. This CTLs response requires several cytotoxic signals or mediators, including granulysin, perforin/granzyme B, and Fas/Fas ligand, to trigger extensive keratinocyte death. In this article, we will discuss the cytotoxic mechanisms of severe cutaneous adverse reactions and their potential applications on therapeutics for this disease.

## 1. Severe Cutaneous Adverse Reactions

Adverse drug reactions (ADRs) are present with a huge variety of phenotypes and frequently affecting the skin. Severe forms of cutaneous adverse reactions are potentially lethal and encompass Stevens-Johnson syndrome (SJS), toxic epidermal necrolysis (TEN), and drug induced eosinophilia and systemic syndrome (DRESS), also known as drug induced hypersensitivity syndrome (DIHS). These conditions are rare but could cause significant morbidity and mortality. Here, we discuss the cytotoxic mechanisms and treatment options of severe cutaneous adverse reactions, with a focus on two related diseases, SJS and TEN.

Stevens-Johnson syndrome (SJS) and toxic epidermal necrolysis (TEN) are considered a spectrum of life-threatening cutaneous diseases, differing only by the degree of epidermal detachment. SJS is defined as the extent of skin detachment involving less than 10% of body surface area (BSA), while TEN is characterized by the detachment greater than 30% of BSA. The severity in-between is categorized as SJS/TEN overlap [1]. Histopathological analyses of skin biopsy specimens from affected areas typically show subepidermal blistering, widespread keratinocyte apoptosis, and full-thickness epidermal necrosis and detachment with a sparse dermal mononuclear infiltrate [2]. The incidence of SJS/TEN is estimated at one to six cases per million inhabitants per year in Europe and the United States [3,4,5,6,7]; however, the mortality rate is 10% for patients with SJS, approximately 30% for patients with SJS/TEN overlap, and almost 50% for patients with TEN [8,9]. Several clinical parameters have been identified as risk factors and incorporated into an illness severity score named SCORTEN (The Severity of Illness Score for Toxic Epidermal Necrolysis), which predicts the mortality [10]. Although microbial infections have occasionally been described as the causes of SJS/TEN, the majority of cases are triggered by drug exposure [1,11,12,13]. To date, over 100 drugs have been associated with this devastating disease [1], among which aromatic anticonvulsants, allopurinol, sulfonamide antibiotics, oxicam nonsteroidal anti-inflammatory drugs, and nevirapine, show a high relative risk [3,6,14,15].

**Figure 1:**
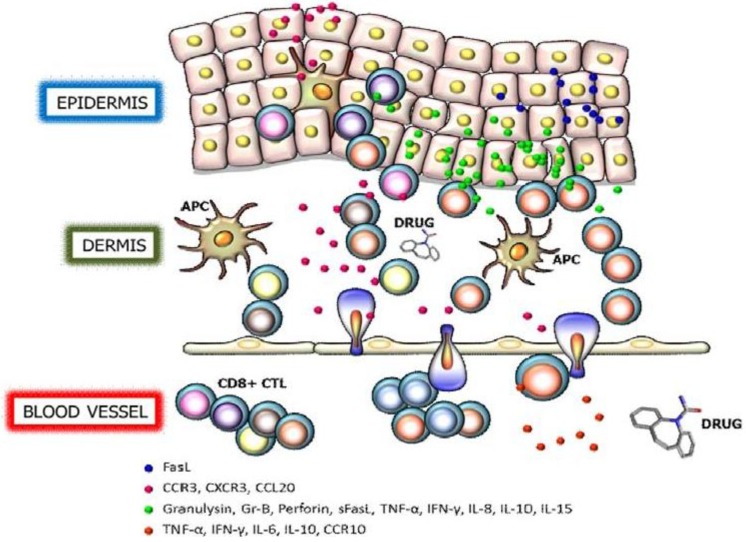
Cytotoxic proteins and cytokines/chemokines in SJS/TEN. Cytotoxic T lymphocytes (CTLs) are activated through the antigen (drug) presentation by the antigen presenting cell (APC) and subsequently carry out the cellular immune reactions directed at keratinocytes in a major histocompatibility class (MHC) I-restricted manner. Upon the activation of CTLs, various cytotoxic proteins, including granulysin, perforin/granzyme B, Fas/Fas ligand, and cytokines/chemokines, are launched to mediate the disseminated keratinocyte death in skin lesions. These toxic signals in turn regulate trafficking, proliferation, and activation of T cells and other immune cells to exaggerate the reaction. The color of CD8+ CTLs is used to illustrate the presence of multiple T cell clones.

Although the pathogenic mechanism of SJS/TEN is not fully understood, current pharmacogenomic studies have demonstrated a correlation of human leukocyte antigen (HLA) genes with drug-induced SJS/TEN [16]. With the identification of specific HLA alleles as the predisposing factor to the condition, it becomes clear that drug-induced SJS and TEN are severe hypersensitivity reactions where cytotoxic T lymphocytes (CTLs) and natural killer (NK) cells are activated and, subsequently, carry out the cellular immune reactions directed at keratinocytes in a major histocompatibility class (MHC) I-restricted manner. Upon the activation of these immunocytes, various cytotoxic signals, including granulysin, perforin/granzyme B, Fas/Fas ligand, and cytokines/chemokines are launched to mediate the disseminated keratinocyte death in skin lesions (Figure 1). Other than the immune etiology, bioactivation of the drug and the genetic variations in genes associated with drug metabolism have also been implicated in the pathogenesis of SJS/TEN or other SCARs [17,18,19]. In this review, functional and therapeutic significance of these cytotoxic mediators in the pathomechanism and patient management of SJS and TEN is discussed. 

## 2. Granulysin

Granulysin is a multifunctional protein present in human CTLs and NK cells, with broad cytolytic activities against a variety of microbes and tumors. It has been studied that granulysin serves as a key molecule responsible for the disseminated keratinocyte death in SJS/TEN [20]. Genome-wide gene expression profiling and immunohistochemical staining of blister cells revealed that granulysin is highly expressed in blister cells of affected individuals. Evidence from experiments using human cells and mice indicated that CD8 + CTLs and NK cells can induce cell death in epidermal keratinocytes through releasing granulysin. Moreover, depletion of granulysin significantly reduced apoptosis of keratinocytes triggered by blister fluids from patients with SJS or TEN. These findings not only support a functional involvement of CTLs, NK cells, and NKT cells in the pathogenesis of SJS and TEN, but also highlight a new cytotoxic mechanism that does not require a direct cellular contact.

Granulysin belongs to the family of saposin-like lipid-interacting proteins and is localized in the granular compartments along with other pore-forming proteins, perforin and granzymes [21]. Two forms of granulysin protein, 9 kDa and 15 kDa, can be found at equivalent levels in human CTLs and NK cells. The 9 kDa form, which is sequestered in the cytolytic granules, is achieved through a proteolytic cleavage at both the amino and carboxyl ends of the 15 kDa precursor, which is constitutively secreted [22]. Crystallography of recombinant granulysin reveals that it is folded as a five-helix bundle stabilized by two conserved intramolecular disulfide bonds [23]. Based on its cationic ampholytic structure, granulysin is able to lyse bacterial membranes which generally are composed of negatively charged lipids. This structural feature renders a scissor-like motion, allowing granulysin to mediate a broad bactericidal activity [24]. In addition, granulysin can kill human cells targeted by CTLs or NK cells by virtue of its permeabilizing effects on mitochondrial and lysosomal membranes. Upon interfering with the membrane of these cellular organelles, cell death pathways are induced through the perturbation of intracellular ion homeostasis [25] and the release of both cytochrome c and apoptosis-inducing factor (AIF) [25,26,27].

In addition to the cytolytic function, both 9 and 15 kDa granulysin have been shown to act as alarmins, endogenous mediators that can induce recruitment and activation of antigen-presenting cells and consequently promote the generation of immune response [28]. Of note, 9 kDa form of granulysin is found to induce the chemotaxis for a variety of immune cells, including T cells, monocytes, and NK cells, and cause an increase in the expression of chemokines (RANTES/CCL5, MCP-1, MCP-3, and MIP-1α/CCL3) and cytokines (IL-1, IL-6, and IFN-α) [29]. Moreover, a recent study indicates that 15 kDa, but not 9 kDa, granulysin is able to activates monocytes to differentiate into immature dendritic cells [30]. These data collectively demonstrate an effect of granulysin on mediating various aspects of immune responses. 

The clinical relevance of multifaceted granulysin is observed not only in SJS/TEN but also in a myriad of diseases [31]. It is reported that granulysin is a vital mediator of damage in a number of cutaneous diseases, including folliculitis [32], psoriasis [33], acne [34], lichen planus [35], and other forms of cutaneous ADRs [36,37]. Noteworthily, numbers of granulysin-positive cells in fixed drug eruptions were similar to those observed in SJS/TEN [36], and serum granulysin levels were elevated in DRESS patients [37]. Furthermore, serum levels of granulysin are markedly heightened and correlated with the severity of graft *vs*. host disease (GVHD) [38], which may mimic SJS and TEN when it presents as similar dermatological manifestations in bone marrow transplant recipients.

## 3. Perforin/Granzyme B

Another hypothetic mechanism underlying the cytotoxicity in SJS/TEN involves perforin and granzyme B, two cytolytic proteins that are released by activated CTLs and NK cells. Increasing levels of perforin, granzyme B, and other toxins, have been observed to be correlated with disease severity of drug hypersensitivity from mild maculopapular rashes to severe TEN [39]. In addition, the inhibition of perforin/granzyme B expression has been shown to attenuate the cytotoxic effect of TEN blister lymphocytes toward keratinocytes, whereas neutralization of Fas/Fas ligand signaling by the anti-Fas monoclonal antibody failed to block cell lysis [40]. 

Exocytosis of cytotoxic compartments is one of the most potent cell death mechanisms employed by activated CTLs and NK cells. Upon lymphocyte recognition of target cells, cytoplasmic granule toxins, predominantly perforin, a membrane-disrupting protein and a family of structurally related serine proteases (granzymes) with various substrate specificities are secreted and together induce apoptosis of the target cells [41]. Perforin is present as a thin ‘key-shaped’ molecule and composed of multiple domains, among which a carboxy-terminal C2 domain mediates initial, Ca^2+^-dependent membrane binding [42,43]. Perforin usually synergizes with granzymes to carry out cytotoxicity. Granzyme B remains the most extensively studied granzyme that induces DNA fragmentation and different death pathways [44]. It is believed that perforin creates pores within the cell membranes, through which the granzymes B can diffuse into the target cell and trigger its killing activity [45]. However, numerous lines of evidence indicate that granzyme B may enter the target cells independently of perforin, either through receptor-mediated endocytosis or macropinocytosis, and is rapidly internalized into endosomes-like vesicles. Subsequently, perforin perturbs endosomal trafficking in the target cell through a process known as endosomolysis, thereby allowing the release of granzyme B into cytosol [46,47,48,49]. This hypothesis is still the topic of vigorous debate as the molecular mechanisms by which perforin enables granzyme B to escape the endosomal compartment into the cytosol have not been clarified yet. In addition, significant amount of perforin has not been shown to be present in the endosome or cytoplasm of the target cell. More recently, a combined theory suggesting that perforin permeabilizes the plasma membrane and this somehow potentiates the uptake of granzymes into an endolytic compartments is proposed [50]. Regardless of which mechanism occurs, all hypotheses assign the key role of perforin to its membranolytic properties, facilitating granzyme B to exit to the cell cytosol, where it can access its substrates.

Once reaching the cytosol, granzyme B is known to induce cell death through the activation of caspase-dependent and -independent pathways. Specifically, granzyme B can directly cleave and activate the caspases [51,52], a large family of endogenous cytosolic proteases that act in a cascade to dismantle the cell. Moreover, granzyme B can trigger caspase activation indirectly by processing of a pro-apoptotic protein Bid into an active form, which then translocates to the mitochondria, interacts with other pro-apoptotic proteins, such as Bax and Bak, and induces mitochondrial outer-membrane permeabilization (MOMP) [53,54]. Subsequently, Bid-mediated mitochondrial damage results in the leakage of pro-apoptotic mitochondrial mediators, such as cytochrome *c*, Smac/Diablo and Omi/HtrA2, into the cytosol to augment the downstream caspase activation [55]. Granzyme B also acts on the mitochondria to induce caspase-independent cell death through the generation of reactive oxygen species (ROS) [56]. Furthermore, granzyme B directly cleaves nuclear lamins, a type of intermediate filaments that mediates the structural function and transcriptional regulation in the cell nucleus, as CTLs induce apoptosis during caspase blockade [57]. This finding provides additional insight into the role of granzyme B in caspase-independent death pathways. Taken together, granzyme B elicits its killing ability through various pathways that may maximize the effectiveness in granule-mediated cell death.

## 4. Fas/Fas Ligand

Fas/Fas ligand (FasL) interaction is another reported mechanism that modulates keratinocyte apoptosis in SJS/TEN and is also of paramount importance for CTL-mediated lysis *in vitro*. The pathophysiological study of Fas and FasL on the epidermal cells of TEN patients revealed that an augmentation of soluble FasL (sFasL) and epidermal FasL expression were observed in the sera and skin biopsy specimens from patients with TEN, respectively, suggesting that sFasL detected in the sera is derived from cleavage of a membrane-bound FasL (mFasL) on the epidermal cells of patients with TEN [58]. Moreover, cell death was abolished by the addition of a FasL-blocking antibody. These data implicate the ligation of FasL expressed by TEN keratinocytes with Fas on adjacent keratinocytes as a critical step in the pathogenesis of SJS/TEN. However, keratinocyte FasL was found to be primarily cytoplasmic *in vivo* and unable to cause apoptosis in a follow-up study [59]. This proposed mechanism was also challenged by the finding that no membrane-bound FasL expression on keratinocytes in TEN patients or in healthy controls can be found, although elevated levels of sFasL in SJS and TEN were detected [60]. Noteworthily, an alternative source of serum sFasL in SJS/TEN was proposed as sFasL levels increased significantly when peripheral blood mononuclear cells (PBMCs) from TEN patients were cultured with the offending drug. Although the involvement of Fas-FasL interactions in mediating keratinocyte death in SJS/TEN was demonstrated in numerous studies, controversy remains as to whether elevated level of sFasL in the TEN sera results from cleavage of mFasL on the epidermal cells or PBMC, as well as whether TEN keratinocytes express lytically active forms of FasL. 

Fas (CD95, also called APO-1) is a trimeric transmembrane protein, belonging to a member of the death receptor (DR) family, a subfamily of the tumor necrosis factor (TNF) receptor superfamily [61]. Ligation of Fas with its cognate ligand, FasL, which is also a TNF related transmembrane molecule [62] and expressed in a far more limited way than the receptor, allows the engagement of receptor and subsequent transduction of the apoptotic signal. Upon the activation, a complex of proteins termed death-inducing signaling complex (DISC) forms and associates with activated Fas [63]. This protein complex encompasses the adaptor, Fas-associated death domain protein (FADD) and pro-apoptotic protease, procaspase-8. The latter is recruited by the former and auto-processed into an active form that is subsequently released from the DISC to the cytoplasm. Activated caspase 8 cleaves various protein substrates in the cytoplasm including procaspase-3 and -7, followed by the activation of nucleases, ultimately leading to the degradation of chromosomal DNA and cell apoptosis [64]. In addition, another Fas-mediated death pathway that is not propagated directly through the caspase cascade has been proposed to be amplified via the mitochondria. In such a paradigm of Fas-induced apoptosis, cleavage of Bid by active caspase-8 mediates the mitochondrial damage, which results in release of cytochrome C [65,66]. Once cytochrome c is released, it interacts with the apoptosis protease activating factor 1 (APAF1) to form the apoptosome, the second initiator complex of apoptosis. The apoptosome unleashes the apoptotic activities by the recruitment and activation of caspase-9, which in turn proteolyzes the downstream effector caspases, caspase-3 and -7, and further triggers a cascade of events, leading to apoptosis [64]. Noteworthily, generation of ROS has also been documented as a key mechanism of apoptosis regulation in Fas-induced cell death and related apoptosis disorders [67].

In addition to the regulation of apoptosis, Fas-FasL interaction has also been shown to play a prominent role in the activation of NF-κB [68,69] and the induction of inflammatory response [70,71,72]. These distinct effects of FasL may result from the functional differences in membrane-anchored and soluble form of this molecule. It is reported that murine sFasL is not apoptotic *in vivo* [73], and under certain circumstances, sFasL may even antagonize the effects of mFasL [74,75]. These diverse activities of Fas suggest that the pathogenic role of epidermal Fas expression in SJS/TEN may be different from that of elevated sFasL detected in the sera.

## 5. Cytokines and Chemokine Receptors

Except for those mentioned above, an overexpression of TNF-α derived from macrophages as well as from keratinocytes was observed in the lesions of TEN, indicating a potential link of TNF-α to extensive necrosis in this disease [76]. TNF-α is a potent cytokine that induces cell apoptosis, cell activation, differentiation, and inflammatory processes [77,78]. Binding of TNF-α to its cell surface receptor triggers apoptosis through DISC-mediated activation of caspase cascade and mitochondrial changes, leading to a series of cytotoxic processes, including generation of free radicals and damage to nuclear DNA by endonucleases [79]. In addition to the apoptotic activities, the pathogenesis of SJS/TEN, in part, is contributed by TNF’s effects on inflammatory response. TNF-α appears to be central to the changes in the vascular endothelial permeability and to the interaction between the leukocytes and vascular endothelium [80,81]. In coordination with the expression of specific cell adhesion molecules, TNF-α is also known to recruit different populations of immunocytes [82,83], which fits the observation that the leukocyte infiltrate remains a key histopathological feature of SJS/TEN. 

Another key cytokine that has been reported to play a key role in SJS/TEN is interferon-γ (IFN-γ) [84]. Although not transmitting apoptotic signal through a conventional death receptor, IFN-γ orchestrates the cytotoxic activities, to some extent, by induction of ROS [85], which is a shared mechanism connecting the involvement of IFN-γ in SJS/TEN with TNF-α and FasL [86]. Moreover, the apoptotic effects of IFN-γ can also be explained by its transcriptional regulation of a variety of genes that are vital for apoptosis, such as TNF-a receptor, Fas/FasL, caspase-1, -4, and -8 [87,88,89,90]. More strikingly, IFN-γ is known to promote antigen presentation and thus stimulate the cell-mediated immunity by upregulation of MHC molecules [85,91,92], further supporting a pathogenic role in SJS/TEN.

Other than TNF-α and IFN-γ, numerous cytokines and chemokine receptors that are responsible for trafficking, proliferation, and activation of T-cells and other immune cells have been found to be elevated in the skin lesions, blister fluids, blister cells, PBMCs, or plasma of SJS/TEN patients. These include IL-2, IL-5, IL-6, IL-10, IL-12, IL-13, IL-15, IL-18, CCR3, CXCR3, CXCR4, and CCR10 [39,84,93,94,95,96]. 

## 6. Therapeutic Interventions of SJS/TEN

With the advances in uncovering the genetic markers of SJS/TEN, the incidence has improved over the past few years with the approval of the genetic screening as a guildline-based test before the use of certain offending drugs [97,98]. Unfortunately, treatment for SJS/TEN remains ineffective and only supportive. Supportive care, including protection of exposed dermis and mucosal surfaces, management of electrolyte and energy intake, pain control, sedation, and detection and treatment of infections, was initiated after admission [99,100]. In addition, prompt withdrawal of causative drugs is believed to be a priority of patient management [101], although early diagnosis of the condition and identification of the offending medication remain a real clinical challenge.

Plasmapheresis and hemodialysis that remove the causative drug, its metabolites or other toxic mediators from the circulation have been used to effectively alleviate the manifestations of SJS/TEN [102,103,104,105]. However, due to the stress associated with this treatment and the lack of formal proof, these strategies at the present time are still debated [11,106].

In addition to elimination of toxins from circulation, many therapy modalities that target those toxic mediators mentioned above have been found to be beneficial for practical management of SJS/TEN. A notable example is the use of systemic corticosteroids in patients with TEN. Corticosteroids, often known as steroids, are an immunosuppressant prescribed for a wide range of conditions. Despite the arguments regarding their efficacy and safety on controlling disease progression [107,108,109], systemic corticosteroids have been the mainstay of the treatment of SJS/TEN for long time, which may be explained by the notion that many toxic mediators involved in the pathogenesis possess not only cytotoxic but also inflammatory effects. Other immunomodulators that have been studied in isolated cases of SJS/TEN include cyclophosphamide [110,111], cyclosporine [112,113,114], *N*-acetylcysteine [115,116], pentoxyfilline [117], and thalidomide [118]. 

Another therapeutic approach directed against the Fas/FasL interaction to treat SJS/TEN has been proposed by using intravenous immunoglobulins (IVIg) [58]. Treatment of this condition with IVIg has been documented in several case series, with conflicting outcomes. Some support the use of IVIg based on reduced mortality rates [119,120,121,122,123], whereas others consider that their efficacy is questionable [124,125,126,127]. A potential clinical trial that targets Fas/FasL interaction through specific blockade, such as anti-Fas (CD95) antibody may further elucidate the pathogenesis of SJS/TEN. 

In addition to targeting the Fas/FasL signaling, several agents have been used to improve the outcome of SJS/TEN through their action on antagonizing the TNF-α pathway. These include TNF inhibitors, pentoxyfilline [117] and thalidomide [118], and an anti-TNF monoclonal antibody, infliximab [128].

## 7. Conclusions

In spite of being uncommon, SJS and TEN have an immense impact on public health by virtue of high mortality. Although the incidence has improved with the identification of the genetic predisposing factors, there is unfortunately no treatment guideline, highlighting the inadequacy of current accepted regimens. The rationale for recent therapeutic interventions of SJS/TEN is mainly derived from empirical evidence regarding our understanding of cytotoxic pathways in keratinocytes and lymphocytes elicited by drug exposure. However, until now, no treatment is specifically directed against granulysin, whose role in epidermal necrolysis has been recognized as a breakthrough in the pathogenesis of SJS/TEN. A reagent blocking the effects of granulysin may be of greater therapeutic value and becomes an urgent need. It is conceivable that a novel treatment for combating granulysin-mediated cell death alone or in combination with existing agents will be beneficial to achieve a better outcome in unfortunate people who suffer from this deadly adverse drug reaction.

## References

[1] Roujeau J.C., Stern R.S. (1994). Severe adverse cutaneous reactions to drugs. N. Engl. J. Med..

[2] Paquet P., Pierard G.E., Quatresooz P. (2005). Novel treatments for drug-induced toxic epidermal necrolysis (Lyell’s syndrome). Int. Arch. Allergy Immunol..

[3] Rzany B., Correia O., Kelly J.P., Naldi L., Auquier A., Stern R. (1999). Risk of Stevens-Johnson syndrome and toxic epidermal necrolysis during first weeks of antiepileptic therapy: A case-control study. Study group of the international case control study on severe cutaneous adverse reactions. Lancet.

[4] French L.E. (2006). Toxic epidermal necrolysis and Stevens Johnson syndrome: Our current understanding. Allergol. Int..

[5] Rzany B., Mockenhaupt M., Baur S., Schroder W., Stocker U., Mueller J., Hollander N., Bruppacher R., Schopf E. (1996). Epidemiology of erythema exsudativum multiforme majus, Stevens-Johnson syndrome, and toxic epidermal necrolysis in Germany (1990–1992): Structure and results of a population-based registry. J. Clin. Epidemiol..

[6] Letko E., Papaliodis D.N., Papaliodis G.N., Daoud Y.J., Ahmed A.R., Foster C.S. (2005). Stevens-Johnson syndrome and toxic epidermal necrolysis: A review of the literature. Ann. Allergy Asthma Immunol..

[7] Ward K.E., Archambault R., Mersfelder T.L. (2010). Severe adverse skin reactions to nonsteroidal antiinflammatory drugs: A review of the literature. Am. J. Health Syst. Pharm..

[8] Pereira F.A., Mudgil A.V., Rosmarin D.M. (2007). Toxic epidermal necrolysis. J. Am. Acad. Dermatol..

[9] Borchers A.T., Lee J.L., Naguwa S.M., Cheema G.S., Gershwin M.E. (2008). Stevens-Johnson syndrome and toxic epidermal necrolysis. Autoimmun. Rev..

[10] Bastuji-Garin S., Fouchard N., Bertocchi M., Roujeau J.C., Revuz J., Wolkenstein P. (2000). SCORTEN: A severity-of-illness *score* for toxic epidermal necrolysis. J. Investig. Dermatol..

[11] Sehgal V.N., Srivastava G. (2005). Toxic epidermal necrolysis (TEN) Lyell’s syndrome. J. Dermatol. Treat..

[12] Mulvey J.M., Padowitz A., Lindley-Jones M., Nickels R. (2007). Mycoplasma pneumoniae associated with Stevens Johnson syndrome. Anaesth Intens. Care.

[13] Forman R., Koren G., Shear N.H. (2002). Erythema multiforme, Stevens-Johnson syndrome and toxic epidermal necrolysis in children: A review of 10 year’ experience. Drug Saf..

[14] Mockenhaupt M., Viboud C., Dunant A., Naldi L., Halevy S., Bouwes-Bavinck J.N., Sidoroff A., Schneck J., Roujeau J.C., Flahault A. (2008). Stevens-Johnson syndrome and toxic epidermal necrolysis: Assessment of medication risks with emphasis on recently marketed drugs. The EuroSCAR-study. J. Invest. Dermatol..

[15] Roujeau J.C., Kelly J.P., Naldi L., Rzany B., Stern R.S., Anderson T., Auquier A., Bastuji-Garin S., Correia O., Locati F. (1995). Medication use and the risk of Stevens-Johnson syndrome or toxic epidermal necrolysis. N. Engl. J. Med..

[16] Bharadwaj M., Illing P., Theodossis A., Purcell A.W., Rossjohn J., McCluskey J. (2012). Drug hypersensitivity and human leukocyte antigens of the major histocompatibility complex. Annu. Rev. Pharmacol. Toxicol..

[17] Sharma A.M., Uetrecht J. (2013). Bioactivation of drugs in the skin: Relationship to cutaneous adverse drug reactions. Drug Metab. Rev..

[18] Yuan J., Guo S., Hall D., Cammett A.M., Jayadev S., Distel M., Storfer S., Huang Z., Mootsikapun P., Ruxrungtham K. (2011). Toxicogenomics of nevirapine-associated cutaneous and hepatic adverse events among populations of African, Asian, and European descent. Aids.

[19] Nayak S., Acharjya B. (2008). Adverse cutaneous drug reaction. Ind. J. Dermatol..

[20] Chung W.H., Hung S.I., Yang J.Y., Su S.C., Huang S.P., Wei C.Y., Chin S.W., Chiou C.C., Chu S.C., Ho H.C. (2008). Granulysin is a key mediator for disseminated keratinocyte death in Stevens-Johnson syndrome and toxic epidermal necrolysis. Nat. Med..

[21] Pena S.V., Hanson D.A., Carr B.A., Goralski T.J., Krensky A.M. (1997). Processing, subcellular localization, and function of 519 (granulysin), a human late T cell activation molecule with homology to small, lytic, granule proteins. J. Immunol..

[22] Hanson D.A., Kaspar A.A., Poulain F.R., Krensky A.M. (1999). Biosynthesis of granulysin, a novel cytolytic molecule. Mol. Immunol..

[23] Anderson D.H., Sawaya M.R., Cascio D., Ernst W., Modlin R., Krensky A., Eisenberg D. (2003). Granulysin crystal structure and a structure-derived lytic mechanism. J. Mol. Biol..

[24] Ernst W.A., Thoma-Uszynski S., Teitelbaum R., Ko C., Hanson D.A., Clayberger C., Krensky A.M., Leippe M., Bloom B.R., Ganz T. (2000). Granulysin, a T cell product, kills bacteria by altering membrane permeability. J. Immunol..

[25] Okada S., Li Q., Whitin J.C., Clayberger C., Krensky A.M. (2003). Intracellular mediators of granulysin-induced cell death. J. Immunol..

[26] Pardo J., Perez-Galan P., Gamen S., Marzo I., Monleon I., Kaspar A.A., Susin S.A., Kroemer G., Krensky A.M., Naval J. (2001). A role of the mitochondrial apoptosis-inducing factor in granulysin-induced apoptosis. J. Immunol..

[27] Kaspar A.A., Okada S., Kumar J., Poulain F.R., Drouvalakis K.A., Kelekar A., Hanson D.A., Kluck R.M., Hitoshi Y., Johnson D.E. (2001). A distinct pathway of cell-mediated apoptosis initiated by granulysin. J. Immunol..

[28] Tewary P., Yang D., de la Rosa G., Li Y., Finn M.W., Krensky A.M., Clayberger C., Oppenheim J.J. (2010). Granulysin activates antigen-presenting cells through TLR4 and acts as an immune alarmin. Blood.

[29] Deng A., Chen S., Li Q., Lyu S.C., Clayberger C., Krensky A.M. (2005). Granulysin, a cytolytic molecule, is also a chemoattractant and proinflammatory activator. J. Immunol..

[30] Clayberger C., Finn M.W., Wang T., Saini R., Wilson C., Barr V.A., Sabatino M., Castiello L., Stroncek D., Krensky A.M. (2012). 15 kDa granulysin causes differentiation of monocytes to dendritic cells but lacks cytotoxic activity. J. Immunol..

[31] Krensky A.M., Clayberger C. (2009). Biology and clinical relevance of granulysin. Tissue Antigens.

[32] Oono T., Morizane S., Yamasaki O., Shirafuji Y., Huh W.K., Akiyama H., Iwatsuki K. (2004). Involvement of granulysin-producing T cells in the development of superficial microbial folliculitis. Br. J. Dermatol..

[33] Raychaudhuri S.P., Jiang W.Y., Raychaudhuri S.K., Krensky A.M. (2004). Lesional T cells and dermal dendrocytes in psoriasis plaque express increased levels of granulysin. J. Am. Acad. Dermatol..

[34] McInturff J.E., Wang S.J., Machleidt T., Lin T.R., Oren A., Hertz C.J., Krutzik S.R., Hart S., Zeh K., Anderson D.H. (2005). Granulysin-derived peptides demonstrate antimicrobial and anti-inflammatory effects against Propionibacterium acnes. J. Investig. Dermatol..

[35] Ammar M., Mokni M., Boubaker S., El Gaied A., Ben Osman A., Louzir H. (2008). Involvement of granzyme B and granulysin in the cytotoxic response in lichen planus. J. Cutan. Pathol..

[36] Schlapbach C., Zawodniak A., Irla N., Adam J., Hunger R.E., Yerly D., Pichler W.J., Yawalkar N. (2011). NKp46+ cells express granulysin in multiple cutaneous adverse drug reactions. Allergy.

[37] Saito N., Abe R., Yoshioka N., Murata J., Fujita Y., Shimizu H. (2012). Prolonged elevation of serum granulysin in drug-induced hypersensitivity syndrome. Br. J. Dermatol..

[38] Nagasawa M., Isoda T., Itoh S., Kajiwara M., Morio T., Shimizu N., Ogawa K., Nagata K., Nakamura M., Mizutani S. (2006). Analysis of serum granulysin in patients with hematopoietic stem-cell transplantation: Its usefulness as a marker of graft-versus-host reaction. Am. J. Hematol..

[39] Posadas S.J., Padial A., Torres M.J., Mayorga C., Leyva L., Sanchez E., Alvarez J., Romano A., Juarez C., Blanca M. (2002). Delayed reactions to drugs show levels of perforin, granzyme B, and Fas-L to be related to disease severity. J. Allergy Clin. Immunol..

[40] Nassif A., Bensussan A., Dorothee G., Mami-Chouaib F., Bachot N., Bagot M., Boumsell L., Roujeau J.C. (2002). Drug specific cytotoxic T-cells in the skin lesions of a patient with toxic epidermal necrolysis. J. Invest. Dermatol..

[41] Trapani J.A., Smyth M.J. (2002). Functional significance of the perforin/granzyme cell death pathway. Nat. Rev. Immunol..

[42] Law R.H., Lukoyanova N., Voskoboinik I., Caradoc-Davies T.T., Baran K., Dunstone M.A., D’Angelo M.E., Orlova E.V., Coulibaly F., Verschoor S. (2010). The structural basis for membrane binding and pore formation by lymphocyte perforin. Nature.

[43] Voskoboinik I., Thia M.C., Fletcher J., Ciccone A., Browne K., Smyth M.J., Trapani J.A. (2005). Calcium-dependent plasma membrane binding and cell lysis by perforin are mediated through its C2 domain: A critical role for aspartate residues 429, 435, 483, and 485 but not 491. J. Biol. Chem..

[44] Lord S.J., Rajotte R.V., Korbutt G.S., Bleackley R.C. (2003). Granzyme B: A natural born killer. Immunol. Rev..

[45] Bots M., Medema J.P. (2006). Granzymes at a glance. J. Cell Sci..

[46] Veugelers K., Motyka B., Goping I.S., Shostak I., Sawchuk T., Bleackley R.C. (2006). Granule-mediated killing by granzyme B and perforin requires a mannose 6-phosphate receptor and is augmented by cell surface heparan sulfate. Mol. Biol. Cell.

[47] Bird C.H., Sun J., Ung K., Karambalis D., Whisstock J.C., Trapani J.A., Bird P.I. (2005). Cationic sites on granzyme B contribute to cytotoxicity by promoting its uptake into target cells. Mol. Cell. Biol..

[48] Motyka B., Korbutt G., Pinkoski M.J., Heibein J.A., Caputo A., Hobman M., Barry M., Shostak I., Sawchuk T., Holmes C.F. (2000). Mannose 6-phosphate/insulin-like growth factor II receptor is a death receptor for granzyme B during cytotoxic T cell-induced apoptosis. Cell.

[49] Froelich C.J., Orth K., Turbov J., Seth P., Gottlieb R., Babior B., Shah G.M., Bleackley R.C., Dixit V.M., Hanna W. (1996). New paradigm for lymphocyte granule-mediated cytotoxicity. Target cells bind and internalize granzyme B, but an endosomolytic agent is necessary for cytosolic delivery and subsequent apoptosis. J. Biol. Chem..

[50] Keefe D., Shi L., Feske S., Massol R., Navarro F., Kirchhausen T., Lieberman J. (2005). Perforin triggers a plasma membrane-repair response that facilitates CTL induction of apoptosis. Immunity.

[51] Martin S.J., Amarante-Mendes G.P., Shi L., Chuang T.H., Casiano C.A., O’Brien G.A., Fitzgerald P., Tan E.M., Bokoch G.M., Greenberg A.H. (1996). The cytotoxic cell protease granzyme B initiates apoptosis in a cell-free system by proteolytic processing and activation of the ICE/CED-3 family protease, CPP32, via a novel two-step mechanism. EMBO J..

[52] Darmon A.J., Nicholson D.W., Bleackley R.C. (1995). Activation of the apoptotic protease CPP32 by cytotoxic T-cell-derived granzyme B. Nature.

[53] Heibein J.A., Goping I.S., Barry M., Pinkoski M.J., Shore G.C., Green D.R., Bleackley R.C. (2000). Granzyme B-mediated cytochrome c release is regulated by the Bcl-2 family members bid and Bax. J. Exp. Med..

[54] Sutton V.R., Davis J.E., Cancilla M., Johnstone R.W., Ruefli A.A., Sedelies K., Browne K.A., Trapani J.A. (2000). Initiation of apoptosis by granzyme B requires direct cleavage of bid, but not direct granzyme B-mediated caspase activation. J. Exp. Med..

[55] Sutton V.R., Wowk M.E., Cancilla M., Trapani J.A. (2003). Caspase activation by granzyme B is indirect, and caspase autoprocessing requires the release of proapoptotic mitochondrial factors. Immunity.

[56] Goping I.S., Sawchuk T., Rieger A., Shostak I., Bleackley R.C. (2008). Cytotoxic T lymphocytes overcome Bcl-2 inhibition: Target cells contribute to their own demise. Blood.

[57] Zhang D., Beresford P.J., Greenberg A.H., Lieberman J. (2001). Granzymes A and B directly cleave lamins and disrupt the nuclear lamina during granule-mediated cytolysis. Proc. Natl. Acad. Sci. USA.

[58] Viard I., Wehrli P., Bullani R., Schneider P., Holler N., Salomon D., Hunziker T., Saurat J.H., Tschopp J., French L.E. (1998). Inhibition of toxic epidermal necrolysis by blockade of CD95 with human intravenous immunoglobulin. Science.

[59] Viard-Leveugle I., Bullani R.R., Meda P., Micheau O., Limat A., Saurat J.H., Tschopp J., French L.E. (2003). Intracellular localization of keratinocyte Fas ligand explains lack of cytolytic activity under physiological conditions. J. Biol. Chem..

[60] Abe R., Shimizu T., Shibaki A., Nakamura H., Watanabe H., Shimizu H. (2003). Toxic epidermal necrolysis and Stevens-Johnson syndrome are induced by soluble Fas ligand. Am. J. Pathol..

[61] Krammer P.H. (2000). CD95’s deadly mission in the immune system. Nature.

[62] Krammer P.H. (1999). CD95(APO-1/Fas)-mediated apoptosis: Live and let die. Adv. Immunol..

[63] Kischkel F.C., Hellbardt S., Behrmann I., Germer M., Pawlita M., Krammer P.H., Peter M.E. (1995). Cytotoxicity-dependent APO-1 (Fas/CD95)-associated proteins form a death-inducing signaling complex (DISC) with the receptor. EMBO J..

[64] Strasser A., Jost P.J., Nagata S. (2009). The many roles of FAS receptor signaling in the immune system. Immunity.

[65] Li H., Zhu H., Xu C.J., Yuan J. (1998). Cleavage of BID by caspase 8 mediates the mitochondrial damage in the Fas pathway of apoptosis. Cell.

[66] Luo X., Budihardjo I., Zou H., Slaughter C., Wang X. (1998). Bid, a Bcl2 interacting protein, mediates cytochrome c release from mitochondria in response to activation of cell surface death receptors. Cell.

[67] Wang L., Azad N., Kongkaneramit L., Chen F., Lu Y., Jiang B.H., Rojanasakul Y. (2008). The Fas death signaling pathway connecting reactive oxygen species generation and FLICE inhibitory protein down-regulation. J. Immunol..

[68] Ahn J.H., Park S.M., Cho H.S., Lee M.S., Yoon J.B., Vilcek J., Lee T.H. (2001). Non-apoptotic signaling pathways activated by soluble Fas ligand in serum-starved human fibroblasts. Mitogen-activated protein kinases and NF-kappaB-dependent gene expression. J. Biol. Chem..

[69] Xiao S., Jodo S., Sung S.S., Marshak-Rothstein A., Ju S.T. (2002). A novel signaling mechanism for soluble CD95 ligand. Synergy with anti-CD95 monoclonal antibodies for apoptosis and NF-kappaB nuclear translocation. J. Biol. Chem..

[70] Peter M.E., Budd R.C., Desbarats J., Hedrick S.M., Hueber A.O., Newell M.K., Owen L.B., Pope R.M., Tschopp J., Wajant H. (2007). The CD95 receptor: Apoptosis revisited. Cell.

[71] Chen J.J., Sun Y., Nabel G.J. (1998). Regulation of the proinflammatory effects of Fas ligand (CD95L). Science.

[72] Wilson N.S., Dixit V., Ashkenazi A. (2009). Death receptor signal transducers: Nodes of coordination in immune signaling networks. Nat. Immunol..

[73] LA O.R., Tai L., Lee L., Kruse E.A., Grabow S., Fairlie W.D., Haynes N.M., Tarlinton D.M., Zhang J.G., Belz G.T. (2009). Membrane-bound Fas ligand only is essential for Fas-induced apoptosis. Nature.

[74] Tanaka M., Suda T., Takahashi T., Nagata S. (1995). Expression of the functional soluble form of human fas ligand in activated lymphocytes. EMBO J..

[75] Knox P.G., Milner A.E., Green N.K., Eliopoulos A.G., Young L.S. (2003). Inhibition of metalloproteinase cleavage enhances the cytotoxicity of Fas ligand. J. Immunol..

[76] Paquet P., Nikkels A., Arrese J.E., Vanderkelen A., Pierard G.E. (1994). Macrophages and tumor necrosis factor alpha in toxic epidermal necrolysis. Arch. Dermatol..

[77] Liu Z.G. (2005). Molecular mechanism of TNF signaling and beyond. Cell Res..

[78] Chavez-Galan L., Arenas-Del Angel M.C., Zenteno E., Chavez R., Lascurain R. (2009). Cell death mechanisms induced by cytotoxic lymphocytes. Cell. Mol. Immunol..

[79] Larrick J.W., Wright S.C. (1990). Cytotoxic mechanism of tumor necrosis factor-alpha. FASEB J. Off. Public. Feder. Am. Soc. Exp. Biol..

[80] Royall J.A., Berkow R.L., Beckman J.S., Cunningham M.K., Matalon S., Freeman B.A. (1989). Tumor necrosis factor and interleukin 1 alpha increase vascular endothelial permeability. Am. J. Physiol..

[81] Norman M.U., Lister K.J., Yang Y.H., Issekutz A., Hickey M.J. (2005). TNF regulates leukocyte-endothelial cell interactions and microvascular dysfunction during immune complex-mediated inflammation. Br. J. Pharmacol..

[82] Hickey M.J., Reinhardt P.H., Ostrovsky L., Jones W.M., Jutila M.A., Payne D., Elliott J., Kubes P. (1997). Tumor necrosis factor-alpha induces leukocyte recruitment by different mechanisms *in vivo* and *in vitro*. J. Immunol..

[83] Kelly M., Hwang J.M., Kubes P. (2007). Modulating leukocyte recruitment in inflammation. J. Allergy Clin. Immunol..

[84] Caproni M., Torchia D., Schincaglia E., Volpi W., Frezzolini A., Schena D., Marzano A., Quaglino P., de Simone C., Parodi A. (2006). Expression of cytokines and chemokine receptors in the cutaneous lesions of erythema multiforme and Stevens-Johnson syndrome/toxic epidermal necrolysis. Br. J. Dermatol..

[85] Schroder K., Hertzog P.J., Ravasi T., Hume D.A. (2004). Interferon-gamma: An overview of signals, mechanisms and functions. J. Leukoc. Biol..

[86] Viard-Leveugle I., Gaide O., Jankovic D., Feldmeyer L., Kerl K., Pickard C., Roques S., Friedmann P.S., Contassot E., French L.E. (2013). TNF-alpha and IFN-gamma are potential inducers of Fas-mediated keratinocyte apoptosis through activation of inducible nitric oxide synthase in toxic epidermal necrolysis. J. Investig. Dermatol..

[87] Chawla-Sarkar M., Lindner D.J., Liu Y.F., Williams B.R., Sen G.C., Silverman R.H., Borden E.C. (2003). Apoptosis and interferons: Role of interferon-stimulated genes as mediators of apoptosis. Apoptosis Int. J. Programmed. Cell Death.

[88] Xu X., Fu X.Y., Plate J., Chong A.S. (1998). IFN-gamma induces cell growth inhibition by Fas-mediated apoptosis: Requirement of STAT1 protein for up-regulation of Fas and FasL expression. Cancer Res..

[89] Tsujimoto M., Yip Y.K., Vilcek J. (1986). Interferon-gamma enhances expression of cellular receptors for tumor necrosis factor. J. Immunol..

[90] Tamura T., Ishihara M., Lamphier M.S., Tanaka N., Oishi I., Aizawa S., Matsuyama T., Mak T.W., Taki S., Taniguchi T. (1997). DNA damage-induced apoptosis and Ice gene induction in mitogenically activated T lymphocytes require IRF-1. Leukemia.

[91] Steimle V., Siegrist C.A., Mottet A., Lisowska-Grospierre B., Mach B. (1994). Regulation of MHC class II expression by interferon-gamma mediated by the transactivator gene CIITA. Science.

[92] Fruh K., Yang Y. (1999). Antigen presentation by MHC class I and its regulation by interferon gamma. Curr. Opin. Immunol..

[93] Paquet P., Paquet F., Al Saleh W., Reper P., Vanderkelen A., Pierard G.E. (2000). Immunoregulatory effector cells in drug-induced toxic epidermal necrolysis. Am. J. Dermatopathol..

[94] Correia O., Delgado L., Barbosa I.L., Campilho F., Fleming-Torrinha J. (2002). Increased interleukin 10, tumor necrosis factor alpha, and interleukin 6 levels in blister fluid of toxic epidermal necrolysis. J. Am. Acad. Dermatol..

[95] Nassif A., Moslehi H., le Gouvello S., Bagot M., Lyonnet L., Michel L., Boumsell L., Bensussan A., Roujeau J.C. (2004). Evaluation of the potential role of cytokines in toxic epidermal necrolysis. J. Invest. Dermatol..

[96] Tapia B., Padial A., Sanchez-Sabate E., Alvarez-Ferreira J., Morel E., Blanca M., Bellon T. (2004). Involvement of CCL27-CCR10 interactions in drug-induced cutaneous reactions. J. Allergy Clin. Immunol..

[97] Su S.C., Chung W.H. (2013). Update on pathobiology in Stevens-Johnson syndrome and toxic epidermal necrolysis. Dermatol. Sin..

[98] Chen P., Lin J.J., Lu C.S., Ong C.T., Hsieh P.F., Yang C.C., Tai C.T., Wu S.L., Lu C.H., Hsu Y.C. (2011). Carbamazepine-induced toxic effects and HLA-B*1502 screening in Taiwan. N. Engl. J. Med..

[99] Chave T.A., Mortimer N.J., Sladden M.J., Hall A.P., Hutchinson P.E. (2005). Toxic epidermal necrolysis: Current evidence, practical management and future directions. Br. J. Dermatol..

[100] Gerull R., Nelle M., Schaible T. (2011). Toxic epidermal necrolysis and Stevens-Johnson syndrome: A review. Crit. Care Med..

[101] Garcia-Doval I., LeCleach L., Bocquet H., Otero X.L., Roujeau J.C. (2000). Toxic epidermal necrolysis and Stevens-Johnson syndrome: Does early withdrawal of causative drugs decrease the risk of death?. Arch. Dermatol..

[102] Sakellariou G., Koukoudis P., Karpouzas J., Alexopoulos E., Papadopoulou D., Chrisomalis F., Skenteris N., Tsakaris D., Papadimitriou M. (1991). Plasma exchange (PE) treatment in drug-induced toxic epidermal necrolysis (TEN). Int. J. Artif. Organs.

[103] Egan C.A., Grant W.J., Morris S.E., Saffle J.R., Zone J.J. (1999). Plasmapheresis as an adjunct treatment in toxic epidermal necrolysis. J. Am. Acad. Dermatol..

[104] Narita Y.M., Hirahara K., Mizukawa Y., Kano Y., Shiohara T. (2011). Efficacy of plasmapheresis for the treatment of severe toxic epidermal necrolysis: Is cytokine expression analysis useful in predicting its therapeutic efficacy?. J. Dermatol..

[105] Yamada H., Takamori K. (2008). Status of plasmapheresis for the treatment of toxic epidermal necrolysis in Japan. Ther. Apher. Dial..

[106] Furubacke A., Berlin G., Anderson C., Sjoberg F. (1999). Lack of significant treatment effect of plasma exchange in the treatment of drug-induced toxic epidermal necrolysis?. Intens. Care Med..

[107] Halebian P.H., Corder V.J., Madden M.R., Finklestein J.L., Shires G.T. (1986). Improved burn center survival of patients with toxic epidermal necrolysis managed without corticosteroids. Ann. Surg..

[108] Revuz J., Penso D., Roujeau J.C., Guillaume J.C., Payne C.R., Wechsler J., Touraine R. (1987). Toxic epidermal necrolysis. Clinical findings and prognosis factors in 87 patients. Arch. Dermatol..

[109] Ruiz-Maldonado R. (1985). Acute disseminated epidermal necrosis types 1, 2, and 3: Study of sixty cases. J. Am. Acad. Dermatol..

[110] Heng M.C., Allen S.G. (1991). Efficacy of cyclophosphamide in toxic epidermal necrolysis. Clinical and pathophysiologic aspects. J. Am. Acad. Dermatol..

[111] Frangogiannis N.G., Boridy I., Mazhar M., Mathews R., Gangopadhyay S., Cate T. (1996). Cyclophosphamide in the treatment of toxic epidermal necrolysis. South. Med. J..

[112] Valeyrie-Allanore L., Wolkenstein P., Brochard L., Ortonne N., Maitre B., Revuz J., Bagot M., Roujeau J.C. (2010). Open trial of ciclosporin treatment for Stevens-Johnson syndrome and toxic epidermal necrolysis. Br. J. Dermatol..

[113] Jarrett P., Rademaker M., Havill J., Pullon H. (1997). Toxic epidermal necrolysis treated with cyclosporin and granulocyte colony stimulating factor. Clin. Exp. Dermatol..

[114] Zaki I., Patel S., Reed R., Dalziel K.L. (1995). Toxic epidermal necrolysis associated with severe hypocalcaemia, and treated with cyclosporin. Br. J. Dermatol..

[115] Redondo P., de Felipe I., de la Pena A., Aramendia J.M., Vanaclocha V. (1997). Drug-induced hypersensitivity syndrome and toxic epidermal necrolysis. Treatment with N-acetylcysteine. Br. J. Dermatol..

[116] Velez A., Moreno J.C. (2002). Toxic epidermal necrolysis treated with N-acetylcysteine. J. Am. Acad. Dermatol..

[117] Sanclemente G., de la Roche C.A., Escobar C.E., Falabella R. (1999). Pentoxyfylline in toxic epidermal necrolysis and Stevens-Johnson syndrome. Int. J. Dermatol..

[118] Wolkenstein P., Latarjet J., Roujeau J.C., Duguet C., Boudeau S., Vaillant L., Maignan M., Schuhmacher M.H., Milpied B., Pilorget A. (1998). Randomised comparison of thalidomide versus placebo in toxic epidermal necrolysis. Lancet.

[119] Stella M., Cassano P., Bollero D., Clemente A., Giorio G. (2001). Toxic epidermal necrolysis treated with intravenous high-dose immunoglobulins: Our experience. Dermatology.

[120] Tristani-Firouzi P., Petersen M.J., Saffle J.R., Morris S.E., Zone J.J. (2002). Treatment of toxic epidermal necrolysis with intravenous immunoglobulin in children. J. Am. Acad. Dermatol..

[121] Prins C., Kerdel F.A., Padilla R.S., Hunziker T., Chimenti S., Viard I., Mauri D.N., Flynn K., Trent J., Margolis D.J. (2003). Treatment of toxic epidermal necrolysis with high-dose intravenous immunoglobulins: Multicenter retrospective analysis of 48 consecutive cases. Arch. Dermatol..

[122] Tan A.W., Thong B.Y., Yip L.W., Chng H.H., Ng S.K. (2005). High-dose intravenous immunoglobulins in the treatment of toxic epidermal necrolysis: An Asian series. J. Dermatol..

[123] Trent J.T., Kirsner R.S., Romanelli P., Kerdel F.A. (2003). Analysis of intravenous immunoglobulin for the treatment of toxic epidermal necrolysis using SCORTEN: The University of Miami Experience. Arch. Dermatol..

[124] Bachot N., Revuz J., Roujeau J.C. (2003). Intravenous immunoglobulin treatment for Stevens-Johnson syndrome and toxic epidermal necrolysis: A prospective noncomparative study showing no benefit on mortality or progression. Arch. Dermatol..

[125] Huang Y.C., Li Y.C., Chen T.J. (2012). The efficacy of intravenous immunoglobulin for the treatment of toxic epidermal necrolysis: A systematic review and meta-analysis. Br. J. Dermatol..

[126] Brown K.M., Silver G.M., Halerz M., Walaszek P., Sandroni A., Gamelli R.L. (2004). Toxic epidermal necrolysis: Does immunoglobulin make a difference?. J. Burn Care Rehabil..

[127] Shortt R., Gomez M., Mittman N., Cartotto R. (2004). Intravenous immunoglobulin does not improve outcome in toxic epidermal necrolysis. J. Burn Care Rehabil..

[128] Fischer M., Fiedler E., Marsch W.C., Wohlrab J. (2002). Antitumour necrosis factor-alpha antibodies (infliximab) in the treatment of a patient with toxic epidermal necrolysis. Br. J. Dermatol..

